# Causal role of vitamin E in atopic dermatitis risk: A Mendelian randomization study

**DOI:** 10.1002/fsn3.4147

**Published:** 2024-03-31

**Authors:** Jian Huang, Youjie Zeng, Yuan Yuan

**Affiliations:** ^1^ Department of Dermatology, Third Xiangya Hospital Central South University Changsha Hunan China; ^2^ Department of Anesthesiology, Third Xiangya Hospital Central South University Changsha Hunan China; ^3^ Department of Pathology, Third Xiangya Hospital Central South University Changsha Hunan China

**Keywords:** antioxidant, atopic dermatitis, causal association, incidence risk, Mendelian randomization, vitamin E

## Abstract

Prior studies suggested that vitamin E might be beneficial in alleviating atopic dermatitis, but confirming a causal link was hindered by limitations such as sample sizes and unaccounted confounders. The present study aimed to clarify this through Mendelian randomization (MR) analysis. GWAS summary statistics was obtained from public databases encompassing a study on vitamin E and two studies related to atopic dermatitis. Two sets of instrumental variables (IVs) were selected using lenient (*p* < 1e‐5) and strict (*p* < 5e‐6) thresholds for separate MR analyses. Inverse variance weighted (IVW) was used as the primary MR method, supplemented by six additional MR methods, and followed by a meta‐analysis to consolidate the impact of vitamin E on atopic dermatitis from two independent studies. Furthermore, various sensitivity tests were performed to assess the reliability of the MR results. A meta‐analysis of IVW analyses deriving from two different atopic dermatitis cohorts under lenient IV selection thresholds demonstrated that vitamin E exhibited a significant lowering risk of atopic dermatitis effect (OR = 0.817, 95% CI: 0.673–0.991, *p* = .041), which was validated under strict IV selection thresholds (OR = 0.822, 95% CI: 0.709–0.954, *p* = .010). In addition, six other MR methods remained parallel to IVW (OR > 1). Multiple sensitivity tests showed that MR analyses were not affected by heterogeneity and horizontal pleiotropy. Overall, this MR study supported vitamin E reducing the risk of atopic dermatitis. Consequently, maintaining an adequate intake of vitamin E could potentially serve as an effective preventive measure against atopic dermatitis.

## Graphical abstract

This study, based on genome‐wide association study data, comprehensively evaluated the causal relationship between vitamin E and atopic dermatitis through Mendelian randomization analysis. The results indicate that increasing vitamin E intake significantly reduce the risk of atopic dermatitis, underscoring the importance of sustaining sufficient daily vitamin E intake for prevention.

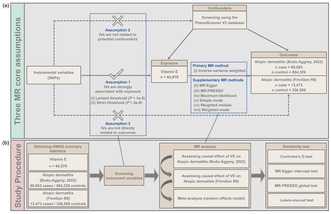

## 1 INTRODUCTION

Atopic dermatitis, commonly referred to as eczema, is a chronic inflammatory skin condition characterized by recurrent episodes that profoundly affect patients' quality of life, posing a significant concern in global public health (Langan et al., 2020). Manifesting through persistent itching, recurring rashes, and diverse severity levels, its complex etiology encompasses genetic predispositions, environmental triggers, immune responses, and disruptions in the skin's protective barriers (Bin & Leung, 2016; Chong et al., 2022). Despite strides in comprehending its mechanisms, managing symptoms and averting flare‐ups remain challenging, highlighting the urgent need for further research. Exploring modifiable risk factors, preventive strategies, and evidence‐based dietary guidelines is crucial to mitigate the incidence and severity of this condition.

Vitamin E, a fat‐soluble antioxidant, has long been acknowledged for its potential in promoting skin health, evident in its presence in various cosmetic products and its protective role against solar radiation damage (Keen & Hassan, 2016). Its antioxidant properties have led to suggestions about its potential in managing chronic inflammatory skin conditions, including atopic dermatitis (Liu et al., 2021). Nevertheless, conflicting findings persist. Studies have shown inconclusive evidence, such as maternal intake of vitamin E during pregnancy not significantly affecting the risk of atopic dermatitis in offspring (Miyake et al., 2010), and no significant association found between vitamin E and atopic dermatitis in children (Nwaru et al., 2014). Consequently, there is insufficient evidence to support recommending vitamin E for atopic dermatitis treatment (Shi & Lio, 2019). Moreover, prior randomized controlled trials (RCTs) were conducted among individuals already diagnosed, leaving uncertainties about the impact of different levels of vitamin E supplementation on the incidence risk of atopic dermatitis. Therefore, the causal relationship between vitamin E and atopic dermatitis remains elusive.

Mendelian randomization (MR) is a statistical method that uses genetic variants as instrumental variables (IVs) to infer causal relationships between an exposure (in this case, vitamin E) and an outcome (atopic dermatitis) (Sanderson et al., 2022). This method has the advantage of minimizing confounding and reducing the possibility of reverse causality, as genetic variants are randomly allocated at conception and are thus unrelated to lifestyle factors (Swerdlow et al., 2016). Single nucleotide polymorphisms (SNPs) are the most utilized form of IVs for implementing MR studies (Sanderson et al., 2022). An important feature of MR studies is their capacity to evaluate the effects of varying levels of lifelong exposure on outcomes (Labrecque & Swanson, 2019). Particularly, in situations where conducting RCTs poses challenges due to cost or ethical constraints, MR studies offer a valuable alternative for assessing causal relationships. The abundance of genome‐wide association study (GWAS) summary statistics has markedly facilitated MR studies, ensuring robust statistical analyses due to substantial sample sizes (Boehm & Zhou, 2022). The present study aimed to determine the causal effect of vitamin E on atopic dermatitis through MR analysis.

## 2 METHODS

### 2.1 Study design

**FIGURE 1:**
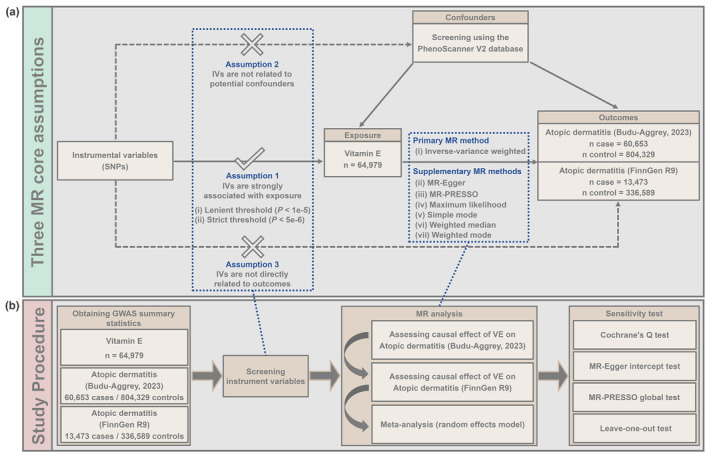
Overview of the present MR study. (a) Three core assumptions of the MR study. (b) The general flow of the present MR study.

In the present study, a two‐sample MR analysis was conducted based on publicly available GWAS summary statistics using the “TwoSampleMR” package (https://mrcieu.github.io/TwoSampleMR/) in R software. This MR study was performed based on three MR core assumptions (Figure 1a): (i) IVs are strongly correlated with exposure; (ii) IVs are not associated with confounding factors; and (iii). IVs are not directly associated with outcome (Boehm & Zhou, 2022). Figure 1b presents the general flowchart of the present MR study. Specifically, one GWAS summary statistic for vitamin E and two GWAS summary statistics for atopic dermatitis were initially obtained from the publicly available databases. Subsequently, IVs representing vitamin E were screened based on three MR core assumptions. Next, the causal effect of vitamin E on atopic dermatitis (from two different outcome sources) was respective estimated and the results were integrated by meta‐analysis with random effects models. Finally, diverse sensitivity tests were performed to evaluate the reliability of the MR results. Subsequent portions of this section will elucidate all the procedures in detail.

### 2.2 Data source for GWAS summary statistics

The GWAS summary statistics for the vitamin E were compiled from the UK Biobank cohort and curated by the MRC‐IEU consortium, which was available for download at the IEU OpenGWAS online platform (https://gwas.mrcieu.ac.uk/datasets/ukb‐b‐6888/) (Ben et al., 2020). This summary statistic was generated based on 64,979 individuals. Subsequently, GWAS summary statistics for atopic dermatitis from two separate cohorts were obtained: (i) a large‐scale GWAS meta‐analysis recently published by Budu‐Aggrey et al. included 60,653 cases and 804,329 controls (available for download in the GWAS Catalog database; https://www.ebi.ac.uk/gwas/studies/GCST90244787) (Budu‐Aggrey et al., 2023); (ii) the FinnGen R9 cohort consisted of 13,473 patients with atopic dermatitis and 336,589 controls (https://r9.finngen.fi/) (Kurki et al., 2023). All included GWAS summary statistics were derived from European individuals.

### 2.3 IVs selection and data harmonization

IVs were screened based on three core assumptions (Figure 1a). To fulfill core assumption 1, all SNPs associated with vitamin E were first screened. Due to the lack of SNPs meeting the genome‐wide significance threshold (*p* < 5e‐8), for the feasibility of analysis, two additional sets of thresholds ([i] lenient threshold: *p* < 1e‐5 and [ii] strict threshold: *p* < 5e‐6) were utilized to screen out two sets of SNPs related to vitamin E. Subsequently, the SNPs in linkage disequilibrium were removed to ensure the independence of each SNP (based on the threshold of *r* ^2^ < 0.001 within 10,000 kb). To fulfill the second core assumption, traits associated with the remaining SNPs were comprehensively examined from the PhenoScanner V2 online platform (Kamat et al., 2019), and SNPs associated with confounders were eliminated. To meet the third core assumption, the association of SNPs with vitamin E should be stronger than their association with atopic dermatitis. Subsequently, the information of the selected SNPs in the GWAS summary statistics for atopic dermatitis was extracted and harmonized, thus ensuring that effect alleles and other alleles were consistent among the summary statistics for exposure and outcome. Furthermore, all palindromic SNPs will be excluded. After all the above screening procedures, the remaining SNPs were included as IVs. Finally, the *F*‐statistics for each IV were computed to evaluate their strength, and only those with *F*‐statistics >10 were able to mitigate the estimation bias arising from weak IVs (Zheng et al., 2017).

### 2.4 Statistical analysis

In the present study, we conducted a series of MR analyses using two distinct IV selection thresholds (*p* < 1e‐5 and *p* < 5e‐6) to evaluate the effect of vitamin E on atopic dermatitis. For each threshold, the effects of vitamin E on atopic dermatitis were assessed using two independent datasets: the Budu‐Aggrey et al. dataset and the FinnGen dataset. The primary MR method applied in this analysis was the inverse variance weighted (IVW) method (Pagoni et al., 2019). Additionally, several complementary MR methods were utilized to reinforce the IVW results, namely MR‐Egger, MR‐PRESSO, maximum likelihood, simple mode, weighted median, and weighted mode (Bowden et al., 2016; Burgess & Thompson, 2017; Hartwig et al., 2017; Milligan, 2003; Verbanck et al., 2018).

Since atopic dermatitis is a binary variable, the results of Mendelian randomization (MR) are presented using odds ratios (OR) and 95% confidence intervals (CI). An OR > 1 indicates that vitamin E increases the risk of atopic dermatitis, while an OR < 1 indicates that vitamin E decreases the risk of atopic dermatitis. The MR results are considered statistically significant only when the meta‐analysis result of the IVW is *p* < .05, and the OR values from all other supplementary MR methods are consistent with the IVW result.

Subsequently, some sensitivity tests were performed. Cochran's Q test was used to assess heterogeneity. MR‐Egger intercept test and MR‐PRESSO global test were used to assess horizontal pleiotropy. Leave‐one‐out test was employed to assess stability.

## 3 RESULTS

### 3.1 Selection of IVs

After eliminating the SNPs in linkage disequilibrium, 31 and 12 independent SNPs associated with vitamin E were identified at the thresholds of *p* < 1e‐5 and *p* < 5e‐6, respectively (Table S1). Next, through the PhenoScanner V2 platform, rs12128707 was found to be associated with educational attainment, rs12165526 with cetraben emollient cream treatment, and rs979218 with epaderm ointment treatment was associated (Table S2). Since they could be potential confounding factors, these three SNPs were excluded from subsequent analyses. Tables S3 and S4 show the SNPs filtering process for obtaining IVs that were used for MR analysis. Ultimately, under the threshold of *p* < 1e‐5, 27 and 26 IVs, respectively, were utilized to assess the causal impact of vitamin E on atopic dermatitis in the Budu‐Aggrey et al. and FinnGen R9 cohorts (Table S5). Meanwhile, under the threshold of *p* < 5e‐6, 10 IVs were used to evaluate the causal impact of vitamin E on atopic dermatitis in both cohorts (Table S6). In addition, the *F*‐statistics of all IVs were >10.

### 3.2 Results of MR analysis

**FIGURE 2:**
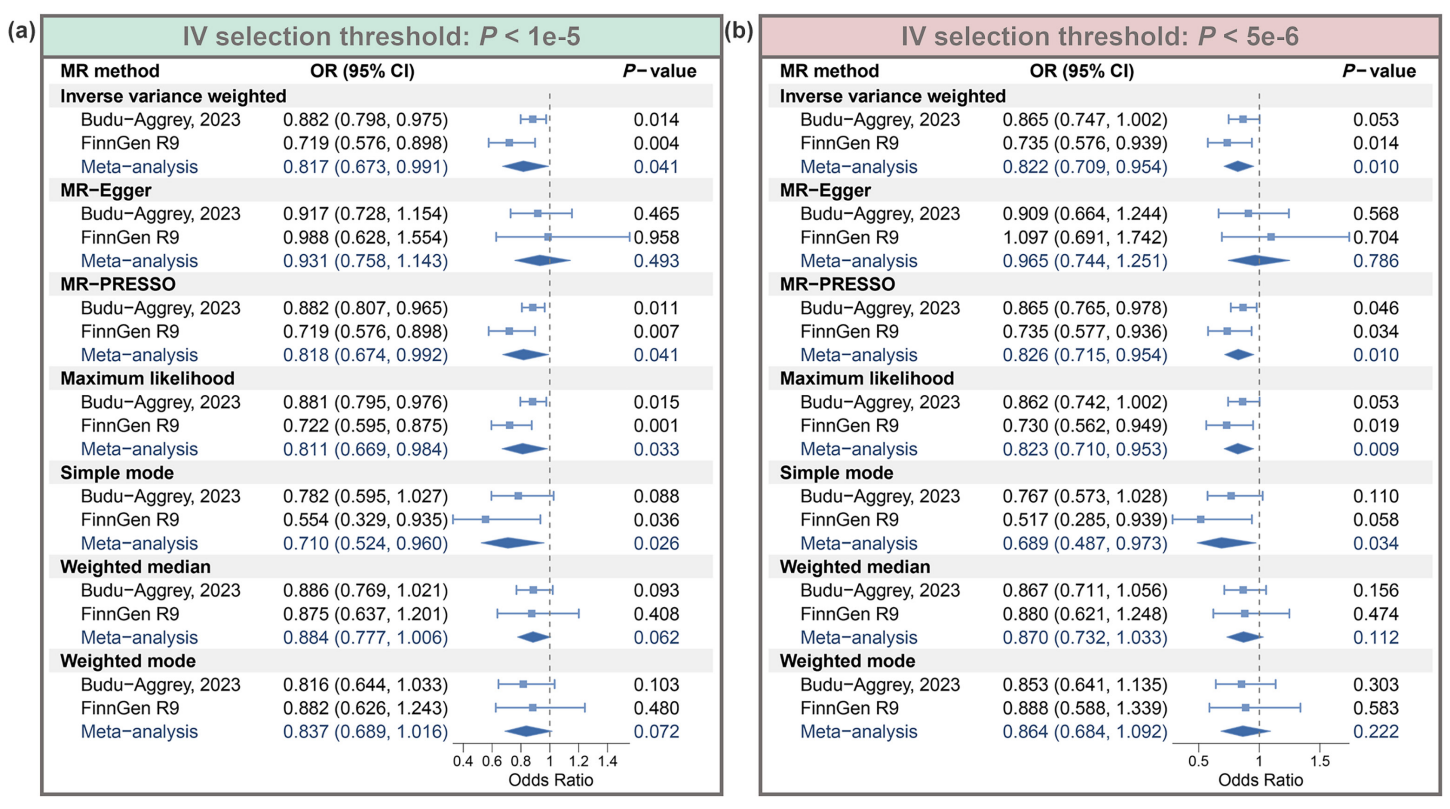
Results of the MR analysis. (a) Results of seven MR analysis approaches based on a lenient IVs selection threshold (*p* < 1e‐5). (b) Results of seven MR analysis approaches based on a strict IVs selection threshold (*p* < 5e‐6).

The forest plot shows the results of this MR study (Figure 2). Based on a lenient IVs selection threshold (*p* < 1e‐5), a meta‐analysis of IVW results in both cohorts showed that vitamin E significantly reduced the risk of atopic dermatitis (OR = 0.817, 95% CI: 0.673–0.991, *p* = .041) (Figure 2a). In addition, the results of MR‐Egger (OR = 0.931, 95% CI: 0.758–1.143, *p* = .493), MR‐PRESSO (OR = 0.818, 95% CI: 0.674–0.992, *p* = .041), maximum likelihood (OR = 0.811, 95% CI: 0.669–0.984, *p* = .033), simple mode (OR = 0.710, 95% CI: 0.524–0.960, *p* = .026), weighted median (OR = 0.884, 95% CI: 0.777–1.006, *p* = .062), and weighted mode (OR = 0.837, 95% CI: 0.689–1.016, *p* = .072) all remained parallel to the IVW results (OR < 1) (Figure 2a). Similarly, based on a strict IVs selection threshold (*p* < 5e‐6), a meta‐analysis of IVW results indicated that vitamin E significantly reduced the risk of atopic dermatitis (OR = 0.822, 95% CI: 0.709–0.954, *p* = .010), while the results of the other six MR methods remained parallel to IVW (Figure 2b). Overall, MR analyses based on both lenient and strict IVs selection threshold demonstrated that vitamin E reduces atopic dermatitis risk.

### 3.3 Results of sensitivity tests

**FIGURE 3:**
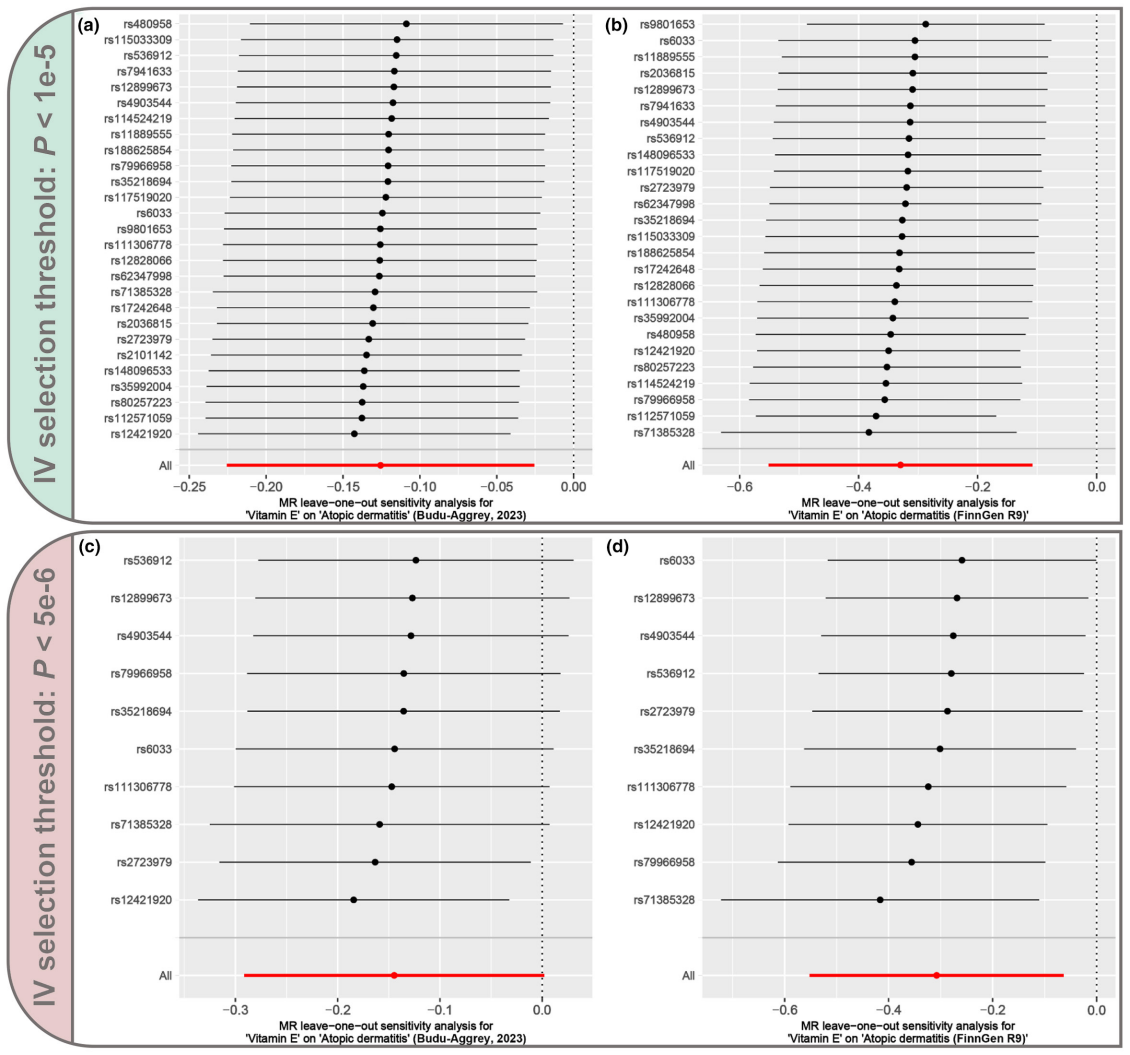
Results of leave‐one‐out sensitivity tests. (a) MR leave‐one‐out sensitivity analysis for “Vitamin E” on “Atopic dermatitis” (Budu‐Aggrey, 2023) based on a lenient IVs selection threshold (*p* < 1e‐5). (b) MR leave‐one‐out sensitivity analysis for “vitamin E” on “atopic dermatitis (FinnGen R9)” based on a lenient IVs selection threshold (*p* < 1e‐5). (c) MR leave‐one‐out sensitivity analysis for “Vitamin E” on “Atopic dermatitis” (Budu‐Aggrey, 2023) based on a strict IVs selection threshold (*p* < 5e‐6). (d) MR leave‐one‐out sensitivity analysis for “Vitamin E” on “Atopic dermatitis (FinnGen R9)” based on a strict IVs selection threshold (*p* < 5e‐6).

Next, various sensitivity tests were performed to assess the reliability of the MR analysis. Cochran's *Q* test showed that there was no significant heterogeneity (*p* > .05) among the IVs screened based on both lenient and strict thresholds (Table 1). In addition, both the MR‐Egger intercept test and the MR‐PRESSO global test showed that MR analyses performed using IVs screened at either threshold were not affected by significant horizontal pleiotropy (*p* > .05) (Table 2). Finally, the leave‐one‐out sensitivity test showed that there were no leading IVs that would significantly impact the overall MR results (Figure 3). Therefore, the sensitivity tests indicated that the MR results were robust with no heterogeneity or horizontal pleiotropy.

**TABLE 1:** Heterogeneity assessed by Cochran's *Q* test.

Outcome data source	Cochran's *Q* test			
	Method	*Q*	Q_df	Q_pval
IV selection threshold: *p* < 1e‐5				
Budu‐Aggrey (2023)	IVW	20.788	26	0.753
	MR Egger	20.656	25	0.712
FinnGen R9	IVW	37.302	25	0.054
	MR Egger	33.857	24	0.087
IV selection threshold: *p* < 5e‐6				
Budu‐Aggrey (2023)	IVW	6.306	9	0.709
	MR Egger	6.183	8	0.627
FinnGen R9	IVW	8.820	9	0.454
	MR Egger	4.811	8	0.778

**TABLE 2:** Horizontal pleiotropy assessed by the MR‐Egger intercept test and the MR‐PRESSO global test.

Outcome data source	MR‐Egger intercept test			MR‐PRESSO global test	
	Intercept	SE	*p*‐Value	RSS obs	*p*‐Value
IV selection threshold: *p* < 1e‐5					
Budu‐Aggrey (2023)	−1.64E‐03	0.005	.719	22.234	.778
FinnGen R9	−1.44E‐02	0.009	.131	40.081	.073
IV selection threshold: *p* < 5e‐6					
Budu‐Aggrey (2023)	−2.32E‐03	0.007	.735	7.444	.753
FinnGen R9	−2.16E‐02	0.011	.080	11.308	.484

## 4 DISCUSSION

This study is the first to use MR analysis to elucidate the role of vitamin E in reducing the risk of atopic dermatitis, thus adding confidence to previous clinical studies suggesting that vitamin E represents a good anti‐atopic dermatitis treatment.

Numerous previous studies have indicated the potential benefits of vitamin E in managing atopic dermatitis. In a randomized controlled trial (RCT) involving 96 subjects with atopic dermatitis, the intake of 400 IU/day of vitamin E was compared to a placebo, revealing a significant improvement in subjective symptoms and serum IgE levels (Tsoureli‐Nikita et al., 2002). Another RCT conducted on 70 participants with mild‐to‐moderate atopic dermatitis, it was found that oral administration of 400 IU of vitamin E significantly improved symptoms and quality of life compared to the placebo group (Jaffary et al., 2015). Nevertheless, these RCTs have been limited by small sample sizes, and could also be influenced by confounding factors that are difficult to eliminate. The present study demonstrated the preventive effect of vitamin E on atopic dermatitis by MR analysis based on large‐sample GWAS summary statistics, thereby strengthening the prior findings.

Whether based on lenient IVs selection thresholds or strict IVs selection thresholds, MR analyses indicated compelling results for vitamin E reducing atopic dermatitis risk. Interestingly, employing a meta‐analysis of IVW results as our primary analytical approach revealed similar findings across two distinct thresholds (OR = 0.817 at the lenient threshold vs. OR = 0.822 at the strict threshold). The result needs to be interpreted rationally. Firstly, vitamin E levels were estimated from participants' dietary questionnaire responses in the UK Biobank cohort, with a median intake of 9.366 mg and a standard deviation of 5.402 mg. Secondly, this MR study simulated the causal effect of a lifelong high level of vitamin E on the risk of atopic dermatitis. Therefore, it can be understood that an increase of 5.402 mg in daily average vitamin E intake throughout life could potentially reduce the risk of developing atopic dermatitis by approximately 18%. Therefore, it is more essential for individuals with high skin disorder risk factors to focus on consuming adequate amounts of vitamin E.

The plausible effect of vitamin E against atopic dermatitis can be attributed to multiple mechanisms. Recent research on atopic dermatitis has highlighted the significance of oxidative stress in contributing to cellular damage and tissue inflammation (Ji & Li, 2016). Reactive oxidants induce lipid peroxidation in epidermal keratinocytes, leading to cellular damage and upregulation of pro‐inflammatory cytokines through the nuclear factor‐κΒ pathway (Kruk & Duchnik, 2014; Wullaert et al., 2011). In addition to its antioxidant properties, vitamin E has demonstrated the ability to mitigate inflammation and enhance the expression of keratinocyte differentiation markers (Hayashi et al., 2012; Kato & Takahashi, 2012). Furthermore, the water‐soluble analog of vitamin E, Trolox, has exhibited the capacity to reduce lipid peroxidation and concurrently increase the activities of key enzymes in keratinocytes, suggesting its potential in combating atopic dermatitis (Gehin et al., 2006).

Overall, this study provides evidence for vitamin E intake for the prevention and treatment of atopic dermatitis. Sources of vitamin E in the diet include various common, specialty, and underutilized edible oils, as well as cereals, legume seeds, animal‐derived products, wild plants, and edible flowers (Shahidi et al., 2021). Therefore, it is crucial to emphasize the consumption of these foods to maintain an adequate daily intake of vitamin E, as deficiencies in this nutrient can lead to inflammation (Roth‐Walter et al., 2024).

Several strengths characterize this study. First, the MR analysis is based on large‐sample GWAS summary statistics, ensuring statistical power. In particular, when using a lenient threshold, a relatively large number of IVs resulted in close to 90% statistical power for IVW (Table S5). Second, the summary statistics for exposure and outcomes are derived from independent European cohorts, reducing the risk of a type I error. Third, two diverse IV selection thresholds, multiple MR methods, and meta‐analysis integration of results for two sets of outcomes were employed, thereby increasing the reliability of the present study.

Nevertheless, this study possesses certain limitations that necessitate clarification. First, this study was conducted based on GWAS summary statistics derived from European individuals, therefore the generalizability of the results to other ethnical populations was uncertain. Second, since the MR study was performed using summary‐level statistics rather than individual‐level statistics, the analysis was restricted to assessing the impact of vitamin E on the entire study population, and stratified analyses were not available. Third, since vitamin E intake was estimated based on participants' responses to a dietary questionnaire, it may be affected by memory bias and response bias.

## 5 CONCLUSION

This MR study provides support for the role of vitamin E in reducing the risk of atopic dermatitis. Consequently, maintaining an adequate intake of vitamin E could potentially serve as an effective preventive measure against atopic dermatitis.

## AUTHOR CONTRIBUTIONS

**Jian Huang:** Conceptualization (lead); data curation (lead); formal analysis (lead); investigation (lead); methodology (lead); validation (lead); visualization (lead); writing – original draft (lead). **Youjie Zeng:** Formal analysis (supporting); investigation (supporting); methodology (supporting); resources (lead); software (lead); validation (supporting); visualization (supporting); writing – review and editing (equal). **Yuan Yuan:** Conceptualization (supporting); project administration (lead); supervision (lead); writing – review and editing (equal).

## FUNDING INFORMATION

This research did not receive any external funding.

## CONFLICT OF INTEREST STATEMENT

The authors declare that they do not have any conflict of interest.

## ETHICAL APPROVAL

Publicly available de‐identified data from participant studies approved by an ethical standards committee were used in this study. Therefore, no additional separate ethical approval was required for this study.

## Supporting information


Table S1.

## Data Availability

The GWAS summary statistics utilized for this MR study are accessible from the IEU OpenGWAS database (https://gwas.mrcieu.ac.uk/), the NHGRI‐EBI GWAS Catalog database (https://www.ebi.ac.uk/gwas/), and the FinnGen database (https://www.finngen.fi/).
